# Future perspectives after the guidelines of degenerative cervical myelopathy: A narrative review^☆^

**DOI:** 10.1016/j.jcot.2025.103104

**Published:** 2025-06-14

**Authors:** Narihito Nagoshi, Yoshiharu Kawaguchi

**Affiliations:** a Department of Orthopaedic Surgery, Keio University School of Medicine, 35 Shinanomachi, Shinjuku Ward, Tokyo, 160-8582, Japan; b Department of Orthopedic Surgery, Faculty of Medicine, University of Toyama, 2630 Sugitani, Toyama, 930-0194, Japan

## Abstract

Degenerative cervical myelopathy (DCM) is the most common cause of spinal cord dysfunction in adults, often resulting from cervical spondylotic myelopathy (CSM) or ossification of the posterior longitudinal ligament (OPLL). As the aging population increases, the prevalence of DCM is expected to rise, making the optimization of treatment strategies crucial. While surgical decompression is widely accepted for moderate to severe cases, the management of mild DCM remains controversial. Some studies report significant neurological improvement with surgery, while others find no difference between surgical and conservative approaches. Current guidelines suggest conservative management may be considered for mild cases, with surgical intervention recommended if symptoms progress or do not respond to non-operative treatment.

Non-surgical approaches such as cervical traction therapy and orthotic treatment have been explored, though their long-term effectiveness remains unclear. Pharmacological treatments, including NSAIDs, muscle relaxants, and corticosteroids, are commonly prescribed for symptomatic relief, yet their effectiveness in treating myelopathy-specific symptoms has not been established. According to current guidelines, patients with a modified Japanese Orthopaedic Association (mJOA) score of 15 or higher are considered suitable candidates for conservative management. However, surgical intervention should be considered if there is evidence of symptom progression.

Surgical strategies for DCM vary based on the severity and location of spinal cord compression. The Japanese CSM and OPLL guidelines have extensively compared different surgical approaches. For CSM treatment, while anterior cervical discectomy and fusion (ACDF) and laminoplasty provide similar neurological recovery, ACDF offers better sagittal alignment but carries a higher risk of reoperation. Comparisons between ACDF and posterior decompression and fusion (PDF) indicate that both procedures yield comparable neurological outcomes, though ACDF has been associated with better patient-reported quality of life. In OPLL patients, anterior surgery may be preferable for those with severe kyphosis and extensive anterior compression, despite an increased risk of complications.

Future research should focus on refining diagnostic tools, optimizing surgical decision-making, and assessing the effectiveness of conservative management strategies. Standardizing intraoperative ultrasonography criteria, evaluating the role of postoperative cervical collar immobilization, and investigating rehabilitation protocols are key areas requiring further study.

## 1 Introduction

Degenerative cervical myelopathy (DCM) is a neurological disorder characterized by spinal cord compression resulting from progressive degeneration or ossification of the posterior longitudinal ligament (OPLL).^1^ As the global population ages, the prevalence of DCM is expected to rise significantly.^2^ According to a World Health Organization (WHO) report, by 2050, 38 % of the global population will be aged 65 years and older.^3^ Given this demographic trend, further elucidation of the pathophysiology and the establishment of more effective treatment strategies for cervical spondylotic myelopathy (CSM) and OPLL—two major forms of DCM—are becoming increasingly critical.

Surgical intervention plays a pivotal role in improving neurological function in patients with DCM. Laminoplasty, a widely utilized decompressive procedure, is known for its technical simplicity and stable surgical outcomes, with numerous studies reporting favorable long-term results.^4^, ^5^, ^6^, ^7^ Furthermore, advances in spinal instrumentation have expanded the indications for cervical fusion procedures, offering improved neurological recovery for patients with spinal instability or extensive OPLL.^8^^,^^9^ However, despite these advancements, significant challenges remain in fully understanding the disease pathology and optimizing treatment strategies.

The authors have been actively involved in the Japanese Guideline Committees for CSM and OPLL and have also contributed to the AOSpine DCM Guideline development, engaging in extensive discussions on treatment strategies for DCM.^10^, ^11^, ^12^ This review aims to highlight key emerging challenges in the field and provide insights into future directions based on existing evidence and clinical experience.

## 2 Treatment options for DCM

### 2.1 Strategies for mild DCM

A previous study determined clinically relevant modified Japanese Orthopedic Association (mJOA) cut-offs score for mild, moderate, and severe myelopathy.^13^ Study findings illustrated an mJOA of 14 as the cut-off between mild and moderate myelopathy, and a mJOA of 11 as the cut-off score between moderate and severe disease. Converting these scores to the JOA score resulted in mild and severe myelopathy being classified as 14.5 or higher and as less than 10, respectively.^14^

Based on the modified JOA score, patients with mild DCM (mJOA ≥15) may be considered for initial conservative management. However, surgical intervention is recommended for patients with moderate (mJOA 12–14) to severe (mJOA ≤11) DCM, or when clinical deterioration is observed during conservative treatment. Clear differentiation of treatment strategies based on disease severity is essential for optimizing patient outcomes and is emphasized in current international guidelines.

Surgical decompression results in substantial neurological recovery among patients with moderate to severe myelopathy.^15^ There is no consensus regarding a suitable treatment strategy for mild DCM. Fehlings et al. reported that patients with mild DCM who underwent surgical intervention significantly improved the mJOA score.^15^ Gulati et al., utilizing data from the Norwegian Registry for Spine Surgery, demonstrated that patients with mild DCM experienced clinically meaningful improvements in patient-reported outcome measures (PROMs) following surgical intervention.^16^ However, a randomized controlled trial (RCT) conducted by Kadanka et al. comparing surgical and conservative treatment for cervical myelopathy who were followed up for 10 years, found no significant difference in the final mJOA score between the two groups.^17^ It should be noted that this study included patients with an mJOA score of 12 or higher, and neither surgical nor conservative treatment resulted in significant improvement in the final mJOA score. Consequently, these findings do not provide definitive evidence supporting the efficacy of conservative treatment for mild DCM. Li et al. retrospectively compared outcomes for patients with a JOA score of at least 13 that received surgical and conservative treatment outcomes over an mean follow-up duration of 34 months. They found no significant differences in JOA score or the Neck Disability Index (NDI) between the groups. Although pre- and post-treatment outcomes were not compared, both treatment modalities improved at least one point in the JOA score, suggesting that conservative treatment may be effective in selected cases.^18^

Based on these findings, both the AOSpine DCM Guideline Committee and the Japanese CSM Guideline Committee recommend that conservative treatment may be considered for patients with mild DCM.^10^^,^^12^ However, they caution that surgical intervention should be pursued in cases where patients demonstrate treatment resistance or progressive symptom deterioration. Indeed, a recent study have reported that more than half of patients with DCM managed nonoperatively over a long-term period experienced neurological deterioration.^19^ Further studies with a well-defined research design focusing exclusively on mild DCM patients are warranted to clarify the comparative efficacy of conservative and surgical treatment approaches.

## 3 Cervical traction therapy

Two institutions have investigated the outcomes of continuous cervical traction therapy. One prospective study included 55 cases of CSM presenting with scores of 13 or higher on the JOA score were treated initially by in-bed Good Samaritan cervical traction without surgery.^20^ After a long-term follow-up period of 78.9 months, they reported no significant improvement in JOA scores. Although 41 patients (75 %) maintained their neurological function until the final follow-up, 14 patients (25 %) experienced deterioration, and 12 of them subsequently underwent surgery.

Kong et al. explored the outcomes of 78 patients with CSM and a JOA score of 13+ who underwent a two-week traction therapy using the Good Samaritan method over a mean follow-up period of 40 months. There were no significant improvements in JOA scores with 21 patients (27 %) experiencing neurological deterioration in whom radiological findings indicated a significant association with severe cervical stenosis and cervical instability.^21^

Together, these findings suggest that variations in the duration of traction and follow-up periods across studies make it difficult to draw definitive conclusions regarding the appropriateness of this treatment modality. Furthermore, no studies have specifically examined the efficacy of intermittent traction therapy, leaving its clinical utility uncertain.

## 4 Orthotic therapy

Matsumoto et al. assessed the outcomes of patients with compressive myelopathy and a JOA score of at least 10 who used a cervical orthosis for a minimum least 8 h daily over 3 months.^22^ Three years following recruitment, of the 52 patients recruited, patients with cervical spondylosis exhibited a slight tendency toward JOA score improvement. Furthermore, 72 % of patients either showed an improvement of at least one point in the JOA score or maintained a score of 15 or higher, indicating a favorable prognosis. However, 10 patients (19 %) experienced neurological deterioration and eventually required surgical intervention. These findings suggest that cervical orthoses may have potential short-term benefits. However, given the absence of a control group that did not receive orthotic treatment, it is difficult to assess the true efficacy of this intervention.

## 5 Pharmacological therapy

Nonsteroidal anti-inflammatory drugs (NSAIDs), muscle relaxants, and corticosteroids are commonly prescribed to manage pain and spastic paresis associated with cervical spondylosis. However, the isolated effectiveness of pharmacological treatment in alleviating paralysis or sensory disturbances associated with DCM has not been elucidated. Although off-label, a study reported the use of oral limaprost alfadex (a prostaglandin E1 analogue) in patients with mild DCM, demonstrating improvements in JOA scores and 10-s tests.^23^ However, as this study did not include a control group, its level of evidence remains low.

## 6 General considerations on the efficacy of conservative treatments

Overall, there are few studies investigating the efficacy of conservative treatments for DCM, and the available studies are limited by small sample sizes. Most of these studies are case series thus yielding a low level of evidence. With regard to the balance between the benefits and risks of conservative treatment, some reports suggest that it may contribute to short-term neurological improvement and symptom stabilization. However, the strength of the evidence supporting these claims is weak, making it difficult to determine its true effectiveness. Moreover, there are no reports evaluating adverse events, quality of life (QOL), or pain outcomes associated with conservative management.

For patients with mild to moderate DCM, the decision to pursue conservative treatment is highly dependent on individual patient preferences and physician recommendations, leading to significant variability in treatment selection. Additionally, whether conservative management provides cost-effective benefits remains unknown due to a lack of relevant studies.

### 6.1 Differences in treatment outcomes based on surgical approach

The Japanese Guidelines for CSM and OPLL have systematically compared and evaluated differences in treatment outcomes among various surgical techniques. In conclusion, each surgical approach presents distinct benefits and risks, and the final decision regarding the most appropriate procedure ultimately relies on the surgeon's clinical judgment.

## 7 CSM: ACDF versus laminoplasty

In a prospective study comparing ACDF with laminoplasty, postoperative outcomes at five years demonstrated superior results for ACDF.^24^ However, in a subsequent follow-up survey conducted ten years postoperatively, the differences between the two procedures were not statistically significant.^25^ Similar findings were observed in retrospective comparative studies.^26^^,^^27^

In the systematic review conducted by the Japanese CSM Guideline Committee, no significant difference was observed between ACDF and laminoplasty in terms of pre-to postoperative JOA score improvement.^28^ However, regarding cervical alignment, sagittal alignment following ACDF was found to be superior to that observed after laminoplasty. The overall incidence of complications was higher in the ACDF group, with a significantly increased rate of repeated surgery. Conversely, patients undergoing laminoplasty reported higher postoperative C5 palsy and cervical pain rates.

However, laminoplasty results in generally poor neurological improvement where there are significant anterior compression lesions and kyphotic cervical alignment.^29^^,^^30^ Furthermore, the incidence of postoperative dysphagia,^31^^,^^32^ re-intubation rates,^33^ and pseudarthrosis rates^34^ are significantly increased where ACDF is performed across three or more segments when compared to fusion of one to two segments. Therefore, the anterior approach may be more suitable for patients with kyphosis and extensive anterior compression lesions while being cognizant of the risk of complications. Accordingly, selecting the most appropriate surgical procedure based on the patient's specific pathology and clinical needs is crucial. However, no studies have controlled for the number of affected segments when comparing the outcomes of the anterior approach and laminoplasty, highlighting an important area for future research.

## 8 CSM: ACDF versus posterior decompression and fusion (PDF)

In previous studies comparing the two surgical procedures,^35^^,^^36^ no significant difference was observed in neurological symptom improvement. However, regarding PROMs, the anterior approach demonstrated superior results in the NDI. Additionally, the anterior approach better preserved cervical alignment than PDF.

The CSM Guideline Committee conducted a systematic review that demonstrated that comparable improvements in neurological symptoms between patients that underwent ACDF and PDF,^9^ but superior NDI outcomes in the ACDF group that could be associated with more postoperative neck complaints, cervical alignment, and the incidence of C5 palsy linked to the posterior approach. However, it is important to note that the anterior approach is routinely selected where there is significant anterior compressive pathology and kyphotic alignment, whereas the posterior approach is commonly performed in multilevel disease. Therefore, future studies comparing surgical outcomes between ACDF and PDF should incorporate analyses that adjust for potential confounding factors in demographic data to ensure accurate and meaningful comparisons.

## 9 OPLL: should patients undergo anterior surgery or posterior surgery?

Previous studies comparing ACDF to laminoplasty for the treatment of OPLL have yielded conflicting findings. Sakai et al. demonstrated comparable neurological outcomes between patients with a preoperatively lordotic cervical alignment or a spinal canal occupation ratio of less than 50 % who underwent either of the two procedures.^37^ Conversely, ACDF yielded significantly better recovery rates among patients with preoperative kyphotic alignment or a spinal canal occupation ratio exceeding 50 % when compared to laminoplasty.

Systematic reviews analyzing surgical outcomes further indicated that functional recovery was significantly greater in patients undergoing anterior surgery, particularly in those with an OPLL occupation ratio exceeding 60 % and kyphotic alignment.^38^^,^^39^ But anterior surgery is linked to a higher risk of major perioperative complications, including dysphagia, hoarseness, cerebrospinal fluid (CSF) leakage, and reconstruction failure. A review article showed complication rates of 21.4 % following ACDF and 12.9 % following laminoplasty.^39^ In particular, dural ossification (DO) identified on CT has been recognized as a useful predictor of CSF leakage. Among the radiographic features, the double-layer sign is considered the most strongly associated with an increased risk of this complication.^40^ Additionally, anterior surgery is undertaken over a longer period and results in higher intraoperative blood loss, and a higher reoperation rate.

Yoshii et al. prospectively compared patients who underwent anterior fusion surgery to those who underwent laminoplasty, study findings illustrated superior functional recovery following anterior surgery.^41^ However, they also noted a higher incidence of perioperative complications, particularly dysphagia and graft-related issues, in the anterior approach. Therefore, its use should be limited to surgeons with extensive experience in the procedure.

Nagoshi et al. compared patients who underwent anterior versus posterior fusion surgeries and reported comparable neurological and functional recovery between the two approaches.^8^ Although the overall incidence of surgical complications was similar, segmental motor paralysis was more frequently observed in the posterior fusion group, while postoperative dysphagia was more common in the anterior surgery group.

## 10 OPLL: should patients undergo posterior laminectomy with fusion or laminoplasty?

Laminoplasty is the preferred surgical treatment for cervical OPLL. There has been an increase in the adoption of PDF following advancements in cervical instrumentation with comparable improvements in neurological function, as evaluated by the cervical JOA score, between the two procedures.^42^^,^^43^

PDF has been shown to yield more favorable outcomes for patients with severe multilevel ossification, segmental instability, or a negative K-line status when compared to laminoplasty.^44^^,^^45^ In addition to achieving effective decompression, PDF contributes to maintaining postoperative cervical alignment, reducing neck pain, and preventing further progression of ossification—complications frequently observed following laminoplasty.^42^^,^^46^

Despite these advantages, PDF is associated with a higher incidence of C5 palsy.^46^ Given these considerations, patients with severe OPLL should undergo posterior laminectomy with fusion. However, careful patient selection and surgical planning are essential to minimize complications and optimize outcomes.

### 10.1 Summary of surgical approach selection

To provide a more integrated perspective, we offer a comparative summary of the major anterior and posterior surgical approaches discussed above. Anterior decompression techniques, including anterior cervical discectomy and corpectomy with fusion, are generally associated with superior restoration of cervical sagittal alignment and favorable neurological recovery, particularly in patients with anterior compressive pathology and kyphotic alignment. However, these procedures are also linked to higher rates of perioperative complications, such as dysphagia, cerebrospinal fluid leakage, and increased reoperation rates. In contrast, posterior procedures, including laminoplasty and posterior decompression with fusion, are commonly utilized for multilevel disease and offer a lower risk of anterior complications. Nevertheless, posterior approaches are more frequently associated with C5 palsy and persistent postoperative neck pain.

In patients with OPLL, surgical decision-making is further influenced by factors such as the canal occupation ratio, K-line status, and the presence of dural ossification. Anterior approaches may be advantageous in cases with a high canal occupation ratio (>60 %) or kyphotic alignment, but require surgical expertise due to the elevated risk of complications. Posterior strategies, particularly laminectomy with fusion, are preferred in patients with multilevel OPLL, segmental instability, or K-line (−) status. Given the anatomical complexity and variable progression of OPLL, a careful, individualized selection of surgical approach remains critical.

This decision should be based on a thorough assessment of each patient's anatomical features, disease severity, and clinical goals. Furthermore, recent studies have demonstrated that postoperative rehabilitation contributes to additional functional improvement, supporting the recommendation of a comprehensive treatment approach beyond surgical intervention alone.

## 11 Utility of intraoperative neuromonitoring in Spine Surgery

A previous study investigating the alert threshold for motor evoked potentials (MEPs) in decompression surgery for DCM reported that defining an MEP alarm point as a reduction to less than 30 % of the control waveform increases both the sensitivity and specificity for predicting postoperative motor deterioration, and is therefore considered clinically useful.^47^ Another study evaluating the utility of intraoperative spinal cord monitoring in 140 surgical cases of CSM demonstrated a significant correlation between intraoperative MEP reductions and postoperative motor deficits.^48^ The authors suggest that the establishment of a standardized checklist for intraoperative response to MEP changes would be beneficial in promoting consistent and timely interventions.

With respect to the surgery for OPLL, Uchida et al. reported that waveform changes and conduction delays in spinal cord evoked potentials (SCEPs) were associated with postoperative outcomes regardless of surgical approach.^49^ This finding suggests that intraoperative spinal cord monitoring may contribute to improved surgical outcomes, including a reduction in postoperative complications. Furthermore, studies on posterior decompression and fusion for cervical OPLL have indicated that spinal cord monitoring is beneficial not only for preventing upper limb motor deficits, but also for detecting intraoperative screw malposition and other iatrogenic neurological injuries.^50^

However, a meta-analysis of postoperative upper limb palsy after cervical decompression procedures reported that although intraoperative spinal cord monitoring may help predict and prevent such complications, it cannot fully eliminate the risk of postoperative upper limb paresis.^51^

## 12 Future directions in DCM management

1. Does Intraoperative Ultrasonography Influence Postoperative Neurological Improvement?

Intraoperative ultrasonographic assessment is frequently employed during cervical surgery; however, its clinical utility remains a topic of debate. Several studies have investigated its potential role in predicting postoperative neurological improvement.

Kimura et al. examined the amplitude of dura mater and spinal cord pulsations when the anterior dura mater was elevated from the posterior vertebral surface during intraoperative ultrasonographic evaluation.^52^ Their findings demonstrated that greater spinal cord pulsation were significantly associated with improved lower limb neurological function. Naruse et al. compared intraoperative ultrasonographic evaluation of spinal cord elevation with postoperative MRI-based assessments to determine which more strongly correlates with postoperative clinical improvement.^53^ Their study suggested that intraoperative ultrasonographic assessment more accurately reflects clinical symptom improvement than MRI-based evaluation. Nakaya et al. investigated the longitudinal changes in intraoperative and postoperative percutaneous ultrasonographic findings and their correlation with clinical symptoms.^54^ Study findings showed that ultrasonography could adequately assess for postoperative decompression status. Mihara et al. identified intraoperative ultrasonographic evidence of anterior spinal cord elevation from the dura as a predictive factor for neurological symptom improvement.^55^ Additionally, in anterior decompression and fusion (ACDF) procedures, intraoperative ultrasonographic evaluation was reported to be highly useful in confirming adequate decompression.^56^

These findings collectively suggest that intraoperative ultrasonography may be a valuable tool for confirming spinal cord decompression. However, there remains a lack of standardized assessment criteria, and its prognostic value in predicting neurological improvement is yet to be firmly established. Future research should focus on developing standardized ultrasonographic evaluation criteria and conducting rigorously designed clinical studies to validate its prognostic utility.

2 Is Postoperative Cervical Collar Immobilization Beneficial for Surgical Outcomes?

Postoperative cervical collar immobilization has been adopted in many institutions following both posterior cervical laminoplasty and ACDF. However, its effectiveness remains uncertain.

To date, only one RCT has evaluated the efficacy of cervical collars following posterior laminoplasty in which 90 patients with CSM were allocated to a postoperative collar-wearing or non-collar-wearing group. Study results demonstrated the absence of significant differences in the QOL, neck pain, and cervical alignment one year postoperatively between the two groups, leading to the conclusion that cervical collars provide no substantial benefit in this context.^57^

For ACDF, three RCTs,^58^, ^59^, ^60^ two observational studies,^61^^,^^62^ and one systematic review^63^ have been published. Abbott et al. investigated 33 cases and compared clinical outcomes at six weeks postoperatively.^58^ They concluded that cervical collar use contributed to reductions in NDI and neck pain; however, long-term outcomes were not reported. Campbell et al. analyzed 257 cases who underwent ACDF whose mean age was 44.3 years and 43.3 years in the collar group and the non-collar group, respectively, a stratification suggesting that the study population mainly consisted of patients with degenerative disc disease or herniation.^59^ They only found a significant difference in the Physical Component Score of the SF-36 at one year, but not in fusion rates or other clinical parameters. Overley et al. examined the effects of collar immobilization among patients with a men age of 50 years following single- and two-level ACDF and found no significant differences in one-year postoperative NDI scores or subsidence of the interbody cage by group.^60^ Similarly, the two observational studies reported no significant differences between groups in terms of NDI or fusion rates at one-year postoperatively.^61^^,^^62^

A systematic review incorporating these studies concluded that postoperative cervical collar use provides minimal clinical benefit in terms of symptom relief or fusion outcomes.^63^ Based on these findings, postoperative collar immobilization following both posterior laminoplasty and ACDF for DCM appears unlikely to offer substantial long-term clinical benefits at one year postoperatively. However, further investigation is warranted regarding its potential short-term benefits, such as postoperative pain reduction and hematoma prevention, regardless of surgical approach. Future clinical studies focusing specifically on these aspects are needed to clarify the role of cervical collar immobilization in postoperative management.

3 Does Rehabilitation Improve Postoperative Outcomes?

Rehabilitation is commonly implemented postoperatively in patients with DCM. However, no large-scale clinical studies have comprehensively examined its contribution to clinical symptom improvement. While physical and occupational therapy, including patient education and functional training, are presumed to provide benefits in symptom management and recovery, variations in healthcare insurance systems and hospitalization duration across different countries may pose challenges in objectively evaluating its efficacy.

Despite the limited evidence of postoperative rehabilitation in accelerating the recovery process, its widespread use in clinical practice suggests that it may contribute to patient satisfaction. Future well-designed studies using a standardized rehabilitation protocol are necessary to elucidate its true impact on postoperative functional outcomes.

4 Can Neuroregenerative and Pharmacological Therapies Improve Outcomes in DCM?

Although surgical decompression remains the standard of care in DCM, many patients continue to experience residual neurological deficits. As such, several neuroprotective and neuroregenerative agents have been explored in preclinical and clinical settings. Riluzole, a glutamate release inhibitor, showed neuroprotective effects in animal models but failed to demonstrate significant functional improvement in human trials, including the multicenter CSM-Protect study.^64^ Nevertheless, subgroup analyses suggested potential benefits in pain relief.

Granulocyte colony-stimulating factor (G-CSF) and prostaglandin E1 analogues such as limaprost have shown neuroprotective and angiogenic effects,^23^^,^^65^ although current evidence is limited to early-phase studies. Further large-scale clinical studies are warranted to validate their safety and efficacy.

5 What is the Role of Spinal Cord Biomarkers in Diagnosis and Prognosis?

Reliable biomarkers are critical for improving diagnostic accuracy, prognostication, and treatment stratification in DCM. CSF levels of neurofilament light chain (NfL), glial fibrillary acidic protein (GFAP), and fatty acid-binding protein 3 (FABP3) have been associated with disease severity and functional outcomes.^66^ Elevated NfL levels correlate with lower extremity dysfunction and greater postoperative improvement, suggesting a potential role as a prognostic biomarker.

Blood-based markers such as interleukin-6 (IL-6) and brain-derived neurotrophic factor (BDNF) are also under investigation. Multiplex serum profiling has shown that a combination of NfL, IL-6, and BDNF may aid in early diagnosis and outcome prediction.^67^

Magnetic resonance imaging (MRI) remains the gold standard for anatomical assessment. T2-weighted hyperintensity, T1-weighted hypointensity, and advanced imaging modalities such as diffusion tensor imaging (DTI) have been correlated with histological damage and functional prognosis.^68^ Novel imaging parameters, including brain functional MRI, are currently being explored to enhance predictive accuracy.^69^

6 How Can PROMs Enhance Clinical Assessment?

The use of PROMs provides a patient-centered perspective that complements objective neurological assessments. The Japanese Orthopaedic Association Cervical Myelopathy Evaluation Questionnaire (JOACMEQ) has demonstrated robust reliability and validity across multiple domains, including upper and lower limb function, bladder control, and QOL.^70^ The Neck Disability Index (NDI) and the SF-36 Health Survey are widely utilized in clinical trials and observational studies to assess pain-related disability and overall health status.^71^ Integrating PROMs into routine clinical practice and clinical trials allows for a more holistic evaluation of treatment efficacy and patient satisfaction.

7 What is the Potential of Artificial Intelligence (AI) in DCM Diagnosis and Prognostication?

AI-driven diagnostic tools are increasingly employed in the field of spinal imaging. Deep learning models based on convolutional neural networks (CNNs) have shown high accuracy in identifying spinal cord compression, canal stenosis, and T2 hyperintensities with performance comparable to expert radiologists.^72^

Khan et al. applied machine learning algorithms to predict functional decline one year after surgical decompression for DCM using a cohort of 757 patients.^73^ The model achieved high predictive accuracy and identified key risk factors, including lower baseline mJOA score, male sex, longer symptom duration, and comorbidities.

## 13 Conclusions

In synthesizing current evidence across surgical and non-surgical management strategies for DCM, our review underscores the complexity of clinical decision-making, which is influenced by disease severity, anatomical factors, patient comorbidities, and evolving imaging and monitoring technologies. A nuanced understanding of these interrelated components is essential for the development of personalized treatment strategies and for advancing evidence-based clinical guidelines.

Future investigations should focus on standardizing assessment methods, such as intraoperative ultrasonography, and refining postoperative management strategies, including the use of cervical collars and rehabilitation protocols. Additionally, rigorous comparative studies controlling for demographic confounders are essential to clarify the relative efficacy of different surgical approaches. A multidisciplinary, evidence-based approach will be key to advancing patient care and optimizing long-term outcomes in DCM management.

## Funding

This research was supported by 10.13039/100009619Japan Agency for Medical Research and Development under Grant Number JP24ym0126118.

## Declaration of competing interest

The authors have no competing interests to disclose.
